# Cross-Sectional Analysis: Waist-to-Hip Ratio and Oxygen Saturation Association in Men Exposed to Long-Term Chronic Intermittent Hypobaric Hypoxia

**DOI:** 10.3390/jcm15072485

**Published:** 2026-03-24

**Authors:** Eduardo Pena, Samia El Alam, Karen Flores, Karem Arriaza, Patricia Siques, Julio Brito, Alexandra Del Río, Isaac Cortes, Mário de Castro

**Affiliations:** 1 High Altitude Medicine Research Center, Arturo Prat University, Iquique 1100000, Chile; eduardopena@unap.cl (E.P.); selalam@unap.cl (S.E.A.); kfloresu@unap.cl (K.F.); karriaza@unap.cl (K.A.); patricia.siques.lee@gmail.com (P.S.); juliobrito28001@gmail.com (J.B.); alexa.delrio.camacho@gmail.com (A.D.R.); 2 Mathematics Department, Engineering Faculty, Atacama University, Copiapó 1530000, Chile; 3 Instituto de Ciências Matemáticas e de Computação, Universidade de São Paulo, São Carlos 13566-590, SP, Brazil; mcastro@icmc.usp.br

**Keywords:** high-altitude, waist-to-hip ratio, oxygen saturation, chronic intermittent hypobaric hypoxia, cardiometabolic risk

## Abstract

**Background/Objectives:** Long-term chronic intermittent hypobaric hypoxia (CIHH) is a common occupational exposure among high-altitude workers, particularly miners in northern Chile. This condition consists of working several days above 2500 m followed by rest at sea level, maintaining this cycle for years, which generates physiological alterations. This study analyzed associations among anthropometric indices and biomedical conditions in miners chronically exposed to long-term CIHH. **Methods**: This study was a cross-sectional analysis of 120 healthy Chilean male miners working at altitudes above 4400 m under a 7-day work/7-day rest schedule. Eligibility required ≥5 years of CIHH exposure and absence of cardiopulmonary disease, hypertension, diabetes, or oxygen therapy use. The assessments at altitude included oxygen saturation (SpO_2_), blood pressure, heart rate, hematological parameters, metabolic parameters, and waist-to-hip ratio (WHR); measurements were obtained 18 h after arrival at altitude. WHR, BMI, SpO_2_, and biomedical variables were collected following standardized procedures. Descriptive statistics and group comparisons were performed with Student’s *t*-test or the Wilcoxon test, with statistical significance set at *p* < 0.05. Normality assumption was assessed using the Shapiro–Wilk test. The association between WHR and SpO_2_ was estimated using linear regression, with WHR scaled so that one unit corresponds to a 0.1-unit increase. Adjusted models included BMI, age, and years working under CIHH. Effect sizes and 95% confidence intervals (CIs) were reported. All statistical analyses were performed in the R programming language. **Results**: Mean SpO_2_ was 89.07 ± 0.50% and mean WHR was 0.94 ± 0.01. In unadjusted comparisons, workers with WHR > 0.94 had lower SpO_2_ than those below the threshold (88.8 ± 0.54 vs. 90.41 ± 0.50; *p* = 0.031). In adjusted models, the WHR–SpO_2_ association was small and imprecise (β per 0.1-unit WHR = −0.67 pp; 95% CI −2.08 to 0.74). Hemoglobin showed an independent association with SpO_2_, while other metabolic variables did not materially contribute. **Conclusions**: SpO_2_ showed a modest inverse association with WHR in long-term CIHH workers. Even small saturation decreases may matter at high altitude. Combined WHR–SpO_2_ monitoring may aid occupational surveillance, though longitudinal studies are needed to establish meaningful risk thresholds.

## 1. Introduction

Exposure to long-term chronic intermittent hypobaric hypoxia (CIHH) is characteristic of populations who work at high altitude under rotating shift systems consisting of workdays at high altitude and rest days at sea level, maintaining this system for years (Long-Term CIHH). This particular hypoxic exposure is represented by a decrease in the partial pressure of oxygen (PaO_2_) due to reduced barometric pressure, which constitutes a physiological condition that challenges human homeostasis [1]. One of the consequences of hypoxic exposure is a reduction in arterial oxygen saturation (SpO_2_), inducing a state of limited tissue oxygenation in aerobic organisms, which leads to cardiovascular, hematological, and metabolic adaptations [2,3,4].

In recent decades, CIHH has gained increasing scientific relevance because it represents a hybrid form of hypoxic exposure that differs substantially from both chronic high-altitude residence and acute hypoxia. Workers repeatedly transition between hypobaric hypoxia and sea-level normoxia, producing unique physiological oscillations that may contribute to cumulative biological stress. Such fluctuations influence autonomic regulation, oxidative balance, and systemic inflammation, making CIHH a distinct occupational model for studying cardiometabolic vulnerability.

Studies have determined that CIHH conditions are associated with oxidative stress, activation of the hypothalamic–pituitary–adrenal axis, increased catecholamine levels, and alterations in adipokine signaling, factors that may disrupt cardiometabolic homeostasis [5]. With respect to metabolic alterations, studies in humans under Long-Term CIHH conditions (above 2500 m; 4 years) have demonstrated elevated high-sensitivity C-reactive protein (hs-CRP) levels and observed a high prevalence of overweight subjects, which was related to the ad libitum availability of food and low physical activity (sedentarism) in this CIHH population, together with reduced maximal aerobic capacity (VO_2_max), thereby establishing a cardiometabolic impairment under this particular hypoxic condition [6].

Additionally, several reports have described that CIHH-exposed workers experience sleep fragmentation, reduced ventilatory efficiency, and increased sympathetic activation—factors that may further modulate metabolic and hematological responses to hypoxia. The cyclical nature of CIHH exposure may also influence appetite-regulatory hormones and energy expenditure, contributing to an obesogenic environment in mining camps. These multidimensional stressors emphasize the importance of evaluating anthropometric markers that capture central adiposity rather than general adiposity alone [5,6].

Based on the above, among the most relevant indicators of cardiometabolic risk is the waist-to-hip ratio (WHR), an anthropometric marker that reflects body fat distribution and whose increase is associated with greater visceral adiposity, insulin resistance, and cardiovascular risk [7,8,9]. Unlike the body mass index (BMI), WHR discriminates abdominal fat accumulation, which is considered more atherogenic, metabolically active, and is recognized as a marker of central adiposity [10]. This variable is particularly relevant to assess in hypoxic environments, as exposure to hypoxia has been shown to modulate abdominal fat distribution through mechanisms mediated by transcription factors, such as the stabilization of hypoxia-inducible factor (HIF) [1,11]. Furthermore, the interaction between hypoxia physiology and visceral adiposity remains insufficiently understood in occupational settings. Reduced SpO_2_ may aggravate oxidative stress, alter lipid handling, and promote chronic low-grade inflammation [4], while excess visceral fat may impair ventilatory mechanics and gas exchange [2], suggesting a potential bidirectional relationship. Nevertheless, empirical evidence describing the direct association between WHR and oxygenation in CIHH-exposed workers remains scarce. Therefore, the aim of this study was to determine the association between WHR, lifestyle, biomedical variables, and anthropometric variables in men under conditions of long-term CIHH.

## 2. Materials and Methods

### 2.1. Subjects and Study Design

**Figure 1:**
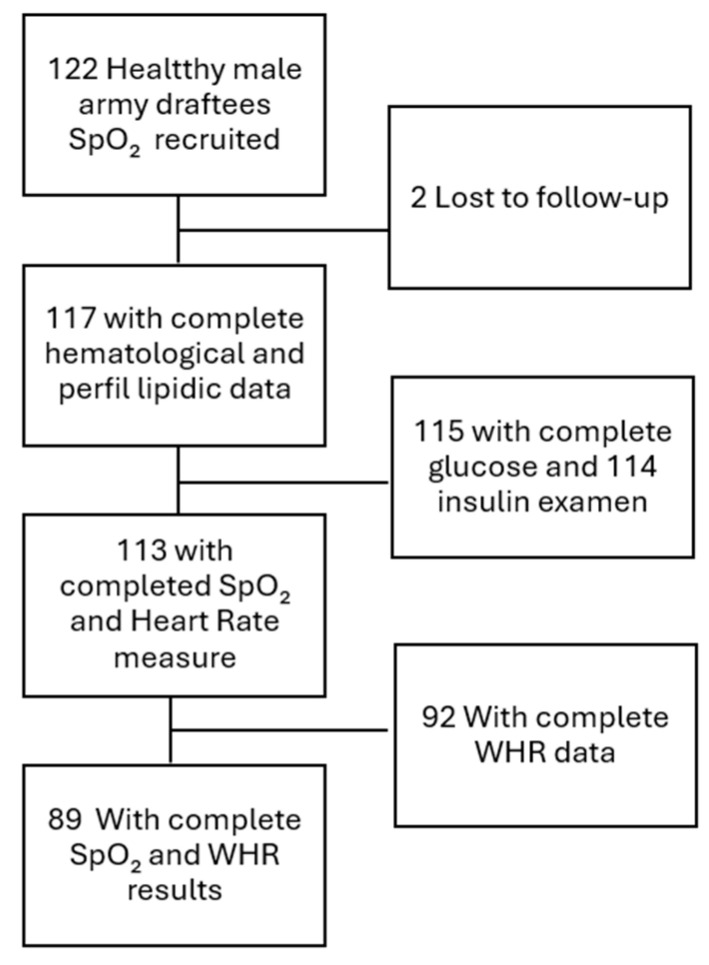
Participant flow diagram for the WHR study, showing eligibility assessment, enrollment, exclusions, missing-data handling, and final analytical samples.

This is a cross-sectional study based on data from a previously published cross-sectional investigation [12]; therefore, temporal ordering between variables cannot be established. The present study evaluates additional associations between WHR and other relevant biomedical variables. The study was conducted in a random sample of 120 healthy native Chilean male miners working in a settlement in northern Chile, located at altitudes over 4400 m (Figure 1). They worked in shifts of 7 days at high altitude, followed by 7 days at sea level. The miners slept at facilities over 3800 m and performed 12 h daytime shifts at the highest altitude, with a 30 min commute from the dormitories to the mine pit. All participants underwent medical examinations and laboratory tests to assess fitness for high-altitude work. Inclusion criteria required working on high-altitude shifts (>4000 m) for more than 5 years (Long-term CIHH) and being in good health without major comorbidities. Absence of diabetes and hypertension was verified through occupational medical examinations, including fasting biochemical testing and repeated blood pressure measurements. Only participants without prior clinical diagnoses and without pharmacological treatment for these conditions were eligible. Individuals with normal-to-high blood pressure (prehypertension) or glucose levels within the upper normal range were not excluded, as these do not constitute established disease and are not disqualifying conditions for high-altitude work under Chilean occupational regulations (Chile, Ministry of Health, Supreme Decree No. 594). Exclusion criteria included diabetes, hypertension, diagnosed obstructive sleep apnea, use of supplemental oxygen in dormitories, and any cardiopulmonary disease. Written informed consent was obtained from all participants in accordance with the Declaration of Helsinki; All participants provided written informed consent at the time of the original study. As the present analysis relies exclusively on previously collected and anonymized data, no new ethical approval was required, in accordance with Chilean Law 20.120 [13]. Measurements were taken at altitude early in the morning, 18 h after arrival, for the beginning of the next 7-day work shift. Assessments were performed after one night of sleep to ensure that participants had completed their first nocturnal exposure at altitude and had reached an initial early-acclimatization state, thereby minimizing transient physiological changes that occur during the first hours after ascent. All participants were evaluated at the same standardized time point to ensure consistency across the sample and to reduce variability due to early acute hypoxic responses. The study followed STROBE guidelines to enhance methodological quality, transparency, and reporting accuracy (see Supplementary Material).

### 2.2. General Data

Data on age (years), weight (kg), height (m), and years working at high altitude under CIHH conditions were collected. BMI was calculated as weight (kg) divided by height squared (m^2^). Waist and hip circumferences (cm) were measured following standardized anthropometric procedures. Waist circumference was measured at the midpoint between the lower margin of the last palpable rib and the top of the iliac crest, and hip circumference at the widest portion of the buttocks. All measurements were performed in triplicate by the same trained personnel using the same instrument to minimize measurement error and inter-observer variability. The waist-to-hip ratio (WHR) was then calculated as waist circumference divided by hip circumference.

### 2.3. Physiological and Hematological Parameters

Oxygen saturation percentage (SpO_2_; %) and heart rate (HR; beats/min) were determined using a finger pulse oximeter (POX050, Mediaid ^®^, Cerritos, CA, USA), which undergoes routine factory calibration. Although direct recalibration at 4400 m is not technically feasible, we minimized altitude-related variability by ensuring proper sensor placement, verifying adequate peripheral perfusion (warm finger, stable waveform), and obtaining readings only after signal stabilization. Three measurements were taken under standardized resting conditions and with the same device model to reduce inter-device variability. Systolic blood pressure (SBP; mmHg) and diastolic blood pressure (DBP; mmHg) were measured twice on the right arm of each participant while seated and after 5 min of rest, using appropriately sized cuffs and calibrated standard mercury sphygmomanometers according to international guidelines [14,15]. The mean of two measurements separated by a 5 min interval was taken as a valid determination of BP and SpO_2_. Hematological variables that were assessed included hematocrit percentage (Hct; %), hemoglobin (HB; g/dL hemoglobin concentration (Hb; g/dL), mean corpuscular volume (MCV; Fentoliters), mean corpuscular hemoglobin (MCH; picograms), mean corpuscular hemoglobin concentration (MCHC; g/dL), basophils (BAS; µL), eosinophils (EOSI; %), lymphocytes (LYM; %), monocytes (MONOC; %), total cholesterol (CHOLEST; mg/dL), high-density lipoproteins (HDL; mg/dL), low-density lipoproteins (LDL; mg/dL), very-low-density lipoproteins (VLDL; mg/dL), triglycerides (TG; mg/dL), blood glucose (GLY; %), and insulin (INSUL; IU). These samples were collected early in the morning after an overnight fast, approximately 18 h after the participants’ arrival at altitude and following their first night of sleep. Fasting status was verified verbally prior to sampling.

### 2.4. Direct Acyclic Graph (DAG)

We developed a directed acyclic graph (DAG) to guide covariate selection, representing hypothesized relations among WHR, SpO_2_, and contextual factors under CIHH. Age, years in CIHH, were specified as potential confounders; hematological adaptation (Hb/Hct) and other downstream physiological measures were considered mediators and were not included in adjustment sets. The direction WHR → SpO_2_ was specified solely as a working assumption for confounder identification and does not imply causality.

### 2.5. Statistical Analysis

Descriptive statistics were calculated for each variable, including the minimum, maximum, mean, quantiles (for SpO_2_ and WHR), and standard error (SE). The assumption of normality was assessed using the Shapiro–Wilk test. When the null hypothesis of normality was rejected, comparisons between groups were performed using the nonparametric Wilcoxon rank-sum test; otherwise, the Student’s *t*-test was applied. Because multiple physiological variables were evaluated simultaneously, the resulting *p*-values were adjusted using the Benjamini–Hochberg false discovery rate (FDR) procedure to control for the risk of type I error.

The magnitude of the difference in SpO_2_ between WHR groups was further quantified using a standardized effect size (Hedges’ g) together with the mean difference and its 95% confidence interval. In addition, a post hoc power analysis was conducted based on the observed effect size and the sample sizes of the two groups to evaluate the statistical power to detect differences in SpO_2_.

All statistical analyses were performed using R statistical software (R version 4.4.3 (2025-02-28 ucrt)) [16] environment, and statistical significance was initially considered at *p* < 0.05.

#### 2.5.1. Multiple Regression Analysis

To evaluate the association between WHR and oxygen saturation (SpO_2_), a multiple linear regression model was fitted with SpO_2_ as the dependent variable. The main predictor of interest was WHR, and the model was adjusted for potential confounding variables, including age, BMI, years of exposure to intermittent chronic hypobaric hypoxia (years_altitude), and several hematological and metabolic variables.

HB was included as the hematological indicator because of its physiological relevance for oxygen transport and adaptation to hypoxic environments. In addition, the model included metabolic covariates such as VLDL, LDL, CHOLEST, and TG.

To facilitate interpretation of the regression coefficient, WHR was rescaled so that effect estimates correspond to a 0.1-unit increase in WHR. Regression results were summarized by reporting the estimated coefficients (β), their standard errors, *p*-values, and 95% confidence intervals. All statistical analyses were performed using R statistical software.

#### 2.5.2. Robust Regression Analysis

The association between WHR and SpO_2_ was evaluated using linear regression analysis, with SpO_2_ as the dependent variable and WHR as the independent variable. Regression coefficients were estimated using the ordinary least squares (OLS) method. The results of the regression analysis were summarized by reporting the estimated coefficients (β), their standard errors, t-values, and corresponding *p*-values. In addition, 95% confidence intervals (95% CIs) were calculated for the regression parameters. To visualize the relationship between WHR and SpO_2_, a scatter plot was constructed, including the fitted regression line and its 95% confidence band, allowing graphical assessment of the data distribution and potential deviations from linearity.

To evaluate whether individual observations exerted an undue influence on the regression results, Cook’s distance was calculated for each observation. Observations with values exceeding the commonly used threshold of 4/n (where n is the sample size) were considered potentially influential. As a sensitivity analysis, a robust regression model was also fitted using M-estimation, which reduces the influence of extreme observations. The regression coefficients obtained from the robust model were compared with those obtained from the ordinary least squares model to assess the stability of the results.

## 3. Results

### 3.1. General Variables

The study group was 120 miners with a mean age of 41.7 ± 0.67 years and a mean exposure to CIHH of 14 years, which represents the long-term CIHH model. Moreover, the BMI of participants included was considered as overweight (BMI: 26.2 kg/m^2^), according to the World Health Organization (WHO) [17]. Table 1 gives a complete overview of the demographic and anthropometric characteristics of the study group. Distributional analysis of the main study variables showed relatively narrow interquartile ranges. For WHR, the first quartile, median, and third quartile were 0.90, 0.94, and 0.96, respectively. For SpO_2_, the corresponding quartiles were 88%, 90%, and 92%. These values indicate that both central adiposity and oxygenation were clustered within a limited range in this cohort, with modest variability around the median. Figure 1 shows the eligibility assessment, enrollment, exclusions, handling, and final analytical samples.

### 3.2. Biomedical Parameters

#### 3.2.1. Hematological Variables

The hemogram of individuals exposed to long-term CIHH reveals several adaptive and potentially maladaptive alterations. Both hematocrit (47.53%) and hemoglobin (16.27 g/dL) are at the upper physiological limits, consistent with compensatory erythropoiesis in response to hypobaric hypoxia, consistent with secondary erythrocytosis driven by sustained hypoxemia (SpO_2_: 89.07%). These findings indicate a compensatory response aimed at enhancing arterial oxygen-carrying capacity. In addition, the MCV (90.52 fL), MCH (30.88 pg^2^), and MCHC (34.19 g/dL) remain within sea-level reference values, suggesting preserved erythropoiesis quality (Table 2).

Although hematological values were compared with sea-level reference ranges, this was performed only for descriptive purposes. Altitude-adjusted reference ranges are highly population-specific and not standardized for intermittently exposed workers such as CIHH miners. Sea-level ranges provide a consistent benchmark and align with previous CIHH studies in Chilean mining populations. Accordingly, hematological values were interpreted within the context of hypobaric hypoxia exposure without applying diagnostic thresholds derived from permanent high-altitude residents.

Leukocyte differentials show lymphocyte percentages (36.33%) at the upper reference limit (20–40%), eosinophils (2.52%) within the normal range (0–4%), and monocytes (4.98%) at the mid-range (2–8%). Basophil counts (0.41 μL) are within sea-level reference ranges (0–300/μL) (Table 2). Collectively, the white blood cell profile suggests no overt infection or inflammation, although subtle shifts in eosinophils and basophils may reflect environmental or immunologic adaptations. Finally, mean blood glucose (89.49 mg/dL) is within normal limits, whereas insulin levels (11.28 IU) are at the lower end of the reference range (10–20 IU); see Table 2.

#### 3.2.2. Lipid Profile

The lipid analysis showed elevated mean triglycerides (174.60 mg/dL) and VLDL cholesterol (34.98 mg/dL), both above the sea-level reference ranges (TG: 30–150 mg/dL; VLDL: 10–35 mg/dL). Mean total cholesterol (193.80 mg/dL) and LDL cholesterol (113.00 mg/dL) remained within acceptable limits, while HDL cholesterol (43.32 mg/dL) was at the lower end of the optimal range (40–60 mg/dL). This pattern suggests a shift toward atherogenic dyslipidemia, potentially increasing cardiovascular risk associated with WHR in men under CIHH conditions (Table 2).

#### 3.2.3. Blood Pressure and Oxygen Saturation

Mean systolic (126.50 mmHg ± 1.06) and diastolic pressures (81.04 mmHg) are within physiological ranges. Finally, oxygen saturation (89.07%) is below sea-level values (95–100%) (Table 2), confirming the hypoxemic stress inherent to high-altitude exposure and its role in driving the hematological and cardiovascular adaptations observed.

### 3.3. Comparative Analysis of Study Variables Between WHR-Defined Risk Groups

The comparative analysis showed that participants classified in the higher-WHR group (WHR > 0.94) had lower oxygen saturation (SpO_2_) compared with those below this threshold. The unadjusted comparison yielded a *p*-value of 0.031; however, after adjustment for multiple comparisons using the Benjamini–Hochberg false discovery rate procedure, this difference was no longer statistically significant (FDR-adjusted *p* = 0.153).

No significant differences were observed for years working in CIHH (*p* = 0.391), systolic blood pressure (SBP; *p* = 0.238), diastolic blood pressure (DBP; *p* = 0.725), or heart rate (HR; *p* = 0.716) between WHR groups (Table 3). The standardized effect size was Hedges’ g = 0.46 (95% CI: 0.04 to 0.88), indicating a small-to-moderate magnitude of the observed difference.

### 3.4. Clinical and Statistical Interpretation of Adjusted Predictors of Oxygen Saturation

In multiple models, the adjusted association between WHR and SpO_2_ was modest and imprecise (β per 0.1-unit increase in WHR = −0.67 percentage points; 95% CI −2.08 to 0.74; *p* = 0.349), suggesting that any cross-sectional relationship between central adiposity and oxygenation is small within this cohort. Given a mean SpO_2_ of approximately 89%, changes of this magnitude are likely to have limited clinical impact at the individual level, although even small decrements may be relevant in workers operating under already reduced baseline oxygenation. Hemoglobin showed an independent negative association with SpO_2_ (β = −0.67 per 1 g/dL; 95% CI −1.31 to −0.02; *p* = 0.044), which is physiologically consistent with compensatory erythropoiesis under sustained hypoxemia and should be interpreted as a marker of hypoxic adaptation rather than a causal driver of reduced oxygen saturation.

The model explained a modest proportion of the variance in SpO_2_ (R^2^ = 0.143), suggesting that additional determinants (e.g., ventilatory control, sleep-disordered breathing, intra-shift variability, and unmeasured lifestyle factors) likely contribute to variability in oxygen saturation (Table 4 and Table 5).

### 3.5. Assessment of Linearity, Data Dispersion, and Influence Diagnostics

**Figure 2:**
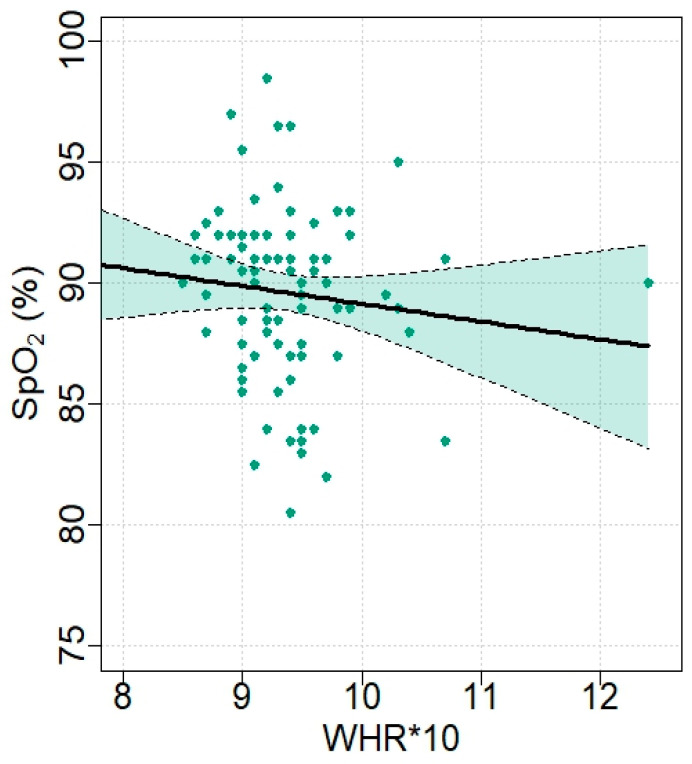
Scatter plot of oxygen saturation (SpO_2_) versus waist-to-hip ratio (WHR), including the fitted linear regression line (black line) and 95% confidence band (green area). The figure illustrates the distribution of individual observations, the modest degree of linear association between WHR and SpO_2_, and the presence of dispersed data points without a strong directional trend.

**Figure 3:**
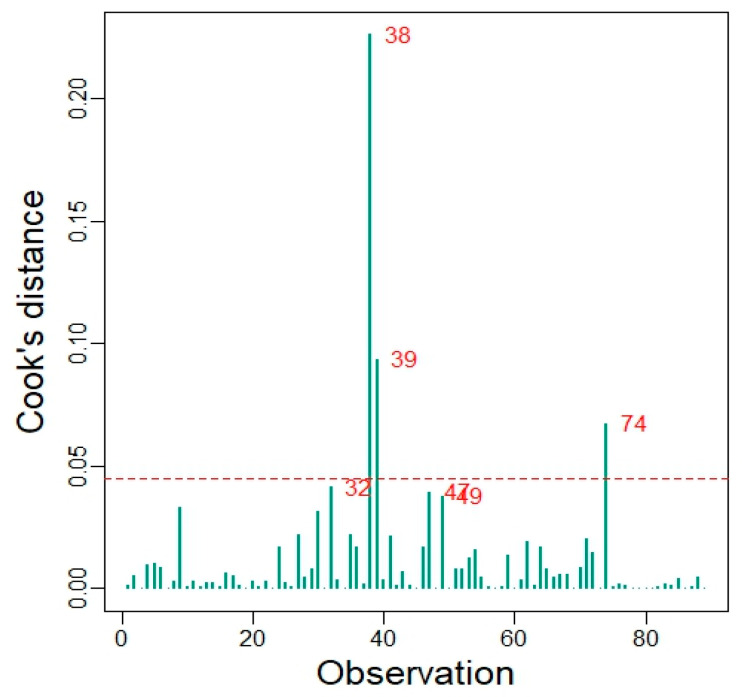
Influence diagnostics based on Cook’s distance for the multivariable linear regression model predicting oxygen saturation (SpO_2_). A small number of observations exhibited higher influence values, but none exceeded conventional thresholds or materially altered the regression estimates, indicating that the association between waist-to-hip ratio (WHR) and SpO_2_ was not driven by outliers.

A scatter plot of SpO_2_ versus WHR, scaled ×10 for interpretability (Figure 2), including the fitted linear regression line and its 95% confidence band, was generated to visualize the distribution of the data, dispersion, and the assumption of linearity. The plot shows substantial scatter around the regression line and no clear linear pattern between WHR and SpO_2_. This visual impression is consistent with the weak and imprecise regression coefficient obtained in the linear model (β per 0.1-unit increase in WHR = −0.73; 95% CI −2.11 to 0.64; *p* = 0.29; R^2^ = 0.013). To evaluate whether individual observations exerted undue influence on the regression estimates, influence diagnostics based on Cook’s distance were examined (Figure 3). A small number of observations showed relatively higher influence values; however, none substantially affected the overall regression results.

As an additional sensitivity analysis, a robust regression model was fitted. The estimated coefficient for WHR remained similar to that obtained using ordinary least squares, with β = −0.73 (SE = 0.69) in the OLS model and β = −0.59 (SE = 0.68) in the robust model. This consistency indicates that the observed association was not driven by influential observations or outliers (Figure 2).

## 4. Discussion

Exposure to long-term CIHH in adult male miners elicits a complex interplay of adaptive and maladaptive physiological responses. In our cohort (mean age, 41.7 years), mean SpO_2_ was 89.1%, consistent with populations living or working at high altitudes [18,19]. Despite this hypoxemia, hematocrit and hemoglobin values remained within reference ranges, indicating a moderate compensatory erythropoietic response comparable to Chilean miners under long-term CIHH [20] and to sea-level natives after eight months of high-altitude exposure [18]. This pattern suggests partial acclimatization under intermittent exposure that supports oxygen transport capacity without progressing to excessive erythrocytosis.

In the immune domain, the lymphocyte percentage (36.3%) approached the upper end of the reference range and aligns with animal studies of chronic intermittent normobaric hypoxia demonstrating increased lymphocyte cytokine production (e.g., IL-2) [21]. These observations warrant caution, as no inflammatory markers or cytokines were measured. Similarly, epidemiological data in young men (17.8 ± 0.7 years) exposed to chronic hypoxia for eight months reported lymphocytosis (42.4% ± 1.2) with concomitant elevations in triglycerides, paralleling the directionality of our cardiometabolic and immune signals [18].

With respect to metabolic outcomes, we observed elevated mean triglycerides (174.6 mg/dL) and increased VLDL fractions, consistent with hypoxia-related disturbances in hepatic lipid handling involving SREBPs [22,23] and hypoxia-inducible factors such as HIF-1α [11]. These pathways are cited solely for physiological context, as we did not perform molecular or inflammatory assessments. Although fasting glucose and insulin were within normal limits, evidence from other hypoxic settings—such as obstructive sleep apnea (OSA) and CIHH animal models—indicates that chronic intermittent hypoxia can impair pancreatic β-cell function, reduce the insulin-to-proinsulin ratio, and increase sympathetic drive, thereby promoting atherogenic dyslipidemia [24,25,26,27]. Notably, some CIHH animal paradigms have reported enhanced insulin sensitivity via AMPK-mediated GLUT4 translocation [25], underscoring that metabolic consequences depend on exposure pattern, duration, and baseline phenotype. In our dataset, glucose–insulin values are descriptive only; without HOMA-IR, no inference regarding insulin sensitivity can be made.

To interpret the association between WHR and SpO_2_, we quantified between-group differences to avoid overstating the effect size. Participants with higher WHR exhibited a statistically significant yet modest reduction in SpO_2_ compared with those below the threshold. Even small decrements may be clinically meaningful in CIHH, where baseline SpO_2_ is already reduced. In line with Pedreros-Lobos et al. [6], our data support oxygenation as a clinically informative marker of cardiometabolic risk in hypoxic environments. However, after multivariable adjustment, the WHR–SpO_2_ association was small and imprecise, consistent with a weak cross-sectional relationship.

WHR may also relate to altered respiratory mechanics. A recent human study reported significant reductions in forced vital capacity (FVC) and forced expiratory volume (FEV) among individuals with high WHR (WHR > 0.85 in females; >1.0 in males), indicating that obesity—even in the absence of overt pulmonary disease—can impair lung function with potential long-term consequences [28]. These considerations are hypothesis-generating in our context, as ventilatory function was not assessed. Moreover, obesity induces complex alterations in respiratory regulation and diaphragm structure that could contribute to oxygenation differences; targeted physical activity—particularly diaphragm-focused training—may improve respiratory efficiency [29], although such mechanisms were not evaluated herein.

Interindividual variability in acclimatization further contextualizes the SpO_2_–WHR relationship. Workers with more than five years of CIHH exposure did not reach the saturation levels reported for Andean Aymara highlanders, suggesting modulation by genetic and epigenetic determinants [30]. These genetic pathways are provided only as background; we did not assess ancestry or molecular markers, and no participant self-identified as Aymara.

Interpretation of erythropoietic adaptation should also consider the healthy-worker effect: individuals with cardiopulmonary disease or reduced tolerance to hypoxia are routinely excluded from high-altitude employment and from participation in such studies, likely limiting variability and yielding an overall healthier cohort.

Regarding external validity, the findings should be interpreted within the context of a relatively homogeneous cohort of healthy adult male miners. The generalizability of associations—particularly those involving oxygen saturation, anthropometry, and hematologic responses—may not extend to women, older workers, or individuals with comorbidities. Sex-specific differences in body composition, hormonal regulation, and cardiopulmonary responses to hypoxia could modify these relationships in women, while aging affects acclimatization capacity and metabolic function. In addition, high-altitude populations with distinct genetic backgrounds or lifelong exposure (e.g., Andean or Tibetan highlanders) may display substantially different adaptations. Caution is therefore warranted when extrapolating beyond the occupational and demographic characteristics of this cohort.

Finally, from translational and public health perspectives, our findings suggest that WHR is not only a cardiometabolic risk marker but also a plausible modulator of acclimatization responses to hypoxia. Although the waist-to-height ratio is gaining traction as a simple screening tool, recent consensus emphasizes that the abdominal adiposity phenotype—captured by WHR—is critical for cardiometabolic risk stratification [31]. Systematic assessment of central adiposity alongside oxygen saturation may better characterize risk under CIHH, inform occupational health policies for high-altitude work, and motivate robust statistical models capable of capturing the complexity of the SpO_2_–WHR relationship. From a clinical and occupational health standpoint, integrating oxygen saturation and central adiposity measures into routine surveillance for workers chronically exposed to CIHH is supported by our findings. Although the adjusted association was modest and not statistically significant, even a 1–2% reduction in SpO_2_ may be operationally relevant at ~4400 m, where baseline saturation is chronically reduced. This pattern suggests that individuals with greater visceral adiposity may be more physiologically strained during high-altitude rotations. The inverse association observed between hemoglobin and SpO_2_ is consistent with adaptive erythropoiesis and should be interpreted as a downstream marker rather than a predictor of oxygenation.

In this cross-sectional analysis, the adjusted WHR–SpO_2_ association was modest and imprecise, indicating that central adiposity showed only a weak relationship with oxygenation among miners exposed to long-term CIHH. Hemoglobin demonstrated an independent inverse association with oxygenation, while additional physiological factors likely contribute to SpO_2_ variability. Collectively, these findings highlight the relevance of anthropometric phenotype as a correlate—rather than a determinant—of oxygenation under CIHH conditions. WHR and SpO_2_ may nonetheless serve as simple, low-burden indicators to support personalized occupational health monitoring. Prospective studies incorporating broader physiological and lifestyle measures will be necessary to refine risk stratification and establish clinically meaningful thresholds.

### Study Limitations

**Figure 4:**
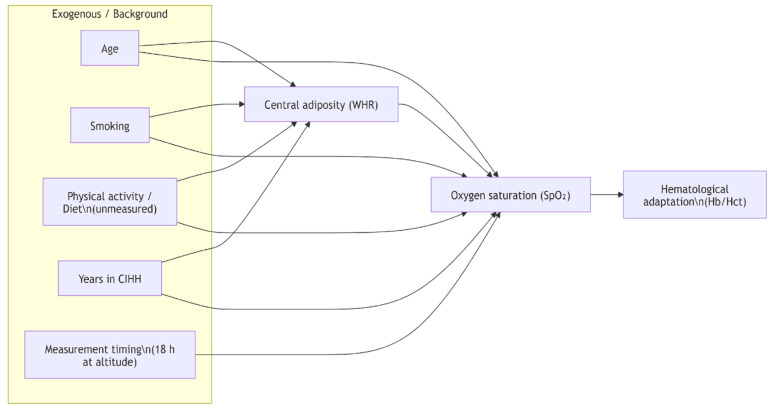
Conceptual DAG for confounding control. The directed acyclic graph depicts hypothesized relationships among central adiposity (waist-to-hip ratio; WHR) and oxygen saturation (SpO_2_) under long-term CIHH exposure. Arrows from Age, Smoking (amount/day; unmeasured), Physical activity/Diet (unmeasured), and Years in CIHH to both WHR and SpO_2_ indicate potential confounding pathways. Measurement timing (18 h at altitude) is shown as a determinant of SpO_2_ standardization but not a confounder of the WHR–SpO_2_ relationship. Hematological adaptation (Hb/Hct) is represented as a downstream mediator of SpO_2_ and should not be adjusted for when estimating the association. The arrow WHR → SpO_2_ reflects the working causal direction used solely for confounder identification and does not imply causality.

This study has several limitations. First, the cross-sectional design precludes causal inference, and all associations should be interpreted descriptively. Second, interpretation of erythropoietic adaptation may be affected by the healthy-worker effect and selection bias: individuals with cardiopulmonary disease, OSA, or reduced tolerance to high-altitude conditions are routinely excluded from employment and were also excluded from this study; consequently, the hematologic profile likely reflects a healthier and more homogeneous subset of the workforce. Third, external validity is limited because the cohort consisted exclusively of healthy adult male miners, restricting generalizability to women, older workers, individuals with comorbidities, or populations with different genetic backgrounds. Fourth, although measurements were standardized, residual acute acclimatization effects and potential pulse-oximeter variability at high altitude cannot be fully excluded. Fifth, a post hoc analysis indicated limited statistical power (59.2%) to detect the observed SpO_2_ differences across WHR strata, implying that non-significant associations should be interpreted with caution. Finally, lifestyle factors and respiratory mechanics were not directly measured; related mechanistic interpretations are therefore hypothesis-generating rather than empirically derived (Figure 4). The absence of validated assessments of physical activity or cardiorespiratory fitness may contribute to residual confounding and should be considered when interpreting these associations.

## 5. Conclusions

Using a cross-sectional approach, this study evaluated the relationship between oxygen saturation and waist-to-hip ratio in men exposed to long-term CIHH. Lower SpO_2_ was found to be associated with higher WHR; however, this relationship must be interpreted as correlational, as the study design does not allow conclusions regarding directionality or causality. These findings add to the growing body of evidence indicating that oxygenation status and central adiposity are interrelated in high-altitude occupational settings, reinforcing the relevance of monitoring both parameters in this population. Although the adjusted association was modest and imprecise, even small differences in SpO_2_ may be operationally meaningful at ~4400 m, where baseline oxygenation is already reduced. While the clinical implications should be considered cautiously, the observed association provides a basis for future research and supports the potential development of preventive strategies and occupational surveillance programs. Prospective studies with broader physiological characterization will be necessary to determine whether WHR-based thresholds can improve risk stratification in CIHH-exposed workers.

## Figures and Tables

**Table 1:** General characteristics and anthropometric variables of the men exposed to long-term chronic intermittent hypobaric hypoxia.

Variables	$\bar{x}$ + SE
Age (years old)	41.69 + 0.68
Years working CIHH (years)	13.95 + 0.50
High (m)	1.72 + 0.01
Weight (Kg)	77.88 + 0.99
BMI (kg/m^2^)	26.22 + 0.28
Hip (cm)	103.7 + 0.96
Waist (cm)	96.98 + 0.93
WHR	0.94 + 0.01

Chronic Intermittent Hypobaric Hypoxia (CIHH); Body Mass Index (BMI); Waist-to-Hip Ratio (WHR).

**Table 2:** Biomedical parameters of men exposed to long-term chronic intermittent hypobaric hypoxia (CIHH).

Biomedical Variables	Min	Max	Mean in CIH Subjects ($\bar{x}$ + SE)	Reference Range at Sea Level
SpO_2_ (%)	47.50	98.50	89.07 + 0.50	95–100
Hct (%)	38.90	55.30	47.53 + 0.31	41–49
Hb (g/dL)	12.50	19.20	16.27 + 0.12	13.0–17.5
MCV (femtoliters)	80.10	99.40	90.52 + 0.32	80–100
MCH (picograms)	28.20	33.70	30.88 + 0.12	27–33
MCHC (g/dL)	31.30	36.60	34.19 + 0.10	32–36
BAS (µL)	0.00	1.00	0.41 + 0.05	0–300
EOSI (%)	0.00	8.00	2.52 + 0.15	0–4
LYM (%)	21.00	55.00	36.33 + 0.67	20–40
MONOC (%)	2.00	10.00	4.98 + 0.17	2–8
CHOLEST (mg/dL)	114.00	276.00	193.80 + 2.95	50–200
HDL (mg/dL)	27.00	82.00	43.32 + 0.84	40–60
LDL (mg/dL)	10.00	264.00	113.00 + 3.18	80–125
VLDL (mg/dL)	12.00	115.00	34.98 + 1.76	10–35
TG (mg/dL)	60.00	575.00	174.60 + 8.76	30–150
GLY (mg/dL)	66.00	188.00	89.49 + 1.36	70–99
INSUL (IU)	0.90	39.20	11.28 + 0.66	10–20
SBP (mmHg)	102.50	156.50	126.50 + 1.06	120–129
DBP (mmHg)	56.00	102.50	81.04 + 0.83	80–84
HR (beats/min)	56.00	112.0	81.61 + 0.95	60–100

Oxygen saturation (SpO_2_; %); Hematocrit (Hct; %); Hemoglobin (Hb); Mean corpuscular volume (MCV); Mean corpuscular hemoglobin (MCH); Mean corpuscular hemoglobin concentration (MCHC); Basophils (BAS); Eosinophils (EOSI); Lymphocytes (LYM); Monocytes (MONOC); Total cholesterol (CHOLEST); High-density lipoproteins (HDL); Low-density lipoproteins (LDL); Very-low-density lipoproteins (VLDL); Triglycerides (TG); Blood glucose (GLY); Insulin (INSUL); Systolic Blood Pressure at altitude (SBP); Diastolic Blood Pressure at altitude (DBP); Heart rate (HR). Values for quantitative variables are mean ($\bar{x}$) + Standard Error (SE), maximum (max) and minimum (min) values. Sea-level reference ranges were obtained from [12].

**Table 3:** Stratification of the measured variables. Comparison of Descriptive Statistics Between Non-Risk and Risk Groups Based on WHR Thresholds.

Variable	Non-Cardiometabolic Risk Factor (WHR < 0.94) Mean ± SE (Range)	Cardiometabolic Risk Factor (WHR ≥ 0.94) Mean ± SE (Range)	*p*-Value	Adjusted *p*-Value (FDR)	Statistical Test
WHR	0.90 ± 0.006 (0.66–0.93)	0.98 ± 0.008 (0.94–1.24)	---	---	---
SpO_2_	90.43 ± 0.502 (82.50–98.50)	88.81 ± 0.540 (80.50–96.50)	0.031 *	0.153	*t*-Test
Years working CIHH	13.26 ± 0.694 (5.00–24.60)	14.04 ± 0.762 (5.00–31.00)	0.391	0.651	Wilcoxon test
SBP	124.17 ± 1.649 (102.50–152.50)	126.87 ± 1.555 (104.00–156.50)	0.238	0.594	*t*-Test
DBP	80.07 ± 1.155 (63.00–97.50)	80.70 ± 1.369 (56.00–102.50)	0.725	0.725	*t*-Test
HR	81.48 ± 1.711 (56.00–109.00)	80.71 ± 1.212 (61.00–95.00)	0.716	0.725	*t*-Test

* *p* < 0.05 Non-Cardiometabolic Risk Factor vs. Cardiometabolic Risk Factor.

**Table 4:** Stratification of the measured variables. Comparison of Descriptive Statistics Between Multivariable Linear Regression Models Assessing Anthropometric, Hematological, and Metabolic Predictors of Oxygen Saturation (SpO_2_) in CIHH-Exposed Miners.

▪ Coefficient	Estimate	SE	t-Value	*p*-Value
▪ Intercept	105.260	9.396	11.202	<2 × 10^−16^
▪ WHR	−0.6693	7.102	−0.942	0.349
▪ Age	0.002	0.058	0.034	0.973
▪ BMI	0.198	0.128	1.551	0.125
▪ Years Altitude	−0.001	0.084	−0.014	0.989
▪ HB	−0.667	0.325	−2.051	0.044
▪ VLDL	0.175	0.270	0.648	0.519
▪ LDL	−0.030	0.048	−0.614	0.541
▪ CHOLEST	0.007	0.048	0.150	0.881
▪ TG	−0.044	0.054	−0.814	0.418
$R^{2}$	0.143			

Waist-to-Hip Ratio (WHR); Hemoglobin (HB); Body Mass Index (BMI); Very-low-density lipoproteins (VLDL); Low-density lipoproteins (LDL); Total cholesterol (CHOLEST); Triglycerides (TG); Standard Error (SE).

**Table 5:** Confidence Intervals for Coefficients of the Multiple Models Predicting Oxygen Saturation (SpO_2_).

▪ Coefficient	2.5%	97.5%
▪ Intercept	86.553	123.967
▪ WHR	−2.083	0.745
▪ Age	−0.114	0.118
▪ BMI	−0.056	0.452
▪ Years Altitude	−0.169	0.166
▪ HB	−1.314	−0.020
▪ VLDL	−0.362	0.712
▪ LDL	−0.126	0.067
▪ CHOLEST	−0.088	0.102
▪ TG	−0.151	0.063

Waist-to-Hip Ratio (WHR); Hemoglobin (HB); Body Mass Index (BMI); Very-low-density lipoproteins (VLDL); Low-density lipoproteins (LDL); Total cholesterol (CHOLEST); Triglycerides (TG); Standard Error (SE).

## Data Availability

Data supporting the findings of this study are available in the GitHub repository; Website: https://github.com/isaaccortes1989/CIHH_Altitud_Medicine (accessed on 19 March 2026).

## Supplementary Materials

The following supporting information can be downloaded at: https://www.mdpi.com/article/10.3390/jcm15072485/s1, Document S1: STROBE.

## Abbreviations

The following abbreviations are used in this manuscript:

CIHH	Chronic intermittent hypobaric hypoxia
SpO_2_	Oxygen Saturation
WHR	Waist-to-Hip Ratio
PaO_2_	Partial Pressure of Oxygen
hs-CRP	High-Sensitivity C-Reactive Protein
VO_2_max	Reduced Maximal Aerobic Capacity
BMI	Body Mass Index
HIF	Hypoxia Inducible Factor
WP	Waist Perimeter
BP	Blood Pressure
HR	Heart Rate
SBP	Systolic Blood Pressure
DBP	Diastolic Blood Pressure
Hct	Hematocrit
Hb	Hemoglobin
MCV	Mean Corpuscular Volume
MCH	Mean Corpuscular Hemoglobin
MCHC	Mean Corpuscular Hemoglobin Concentration
BAS	Basophils
EOSI	Eosinophils
LYM	Lymphocytes
MONOC	Monocytes
CHOLEST	Total Cholesterol
HDL	High-Density Lipoproteins
LDL	Low-Density Lipoproteins
VLDL	Very-Low-Density Lipoproteins
TG	Triglycerides
GLY	Blood Glucose
INSUL	Insulin
SE	Standard Error
IL-2	Interleukin 2
SREBPs	Sterol Regulatory Element-Binding Proteins
OSA	Obstructive Sleep Apnea
GLUT4	Glucose Transporter Type 4
AMPK	AMP-activated Protein Kinase
