# Supplementary material for: Cross-Sectional Analysis: Waist-to-Hip Ratio and Oxygen Saturation Association in Men Exposed to Long-Term Chronic Intermittent Hypobaric Hypoxia
Source: J Clin Med. 2026 Mar 24;15(7):2485. doi: 10.3390/jcm15072485 (PMC13073029; doi:10.3390/jcm15072485)
Supplement: Supplementary file 1 [file jcm-15-02485-s001.zip › jcm-4182312-supplementary.pdf]

STROBE Statement—Checklist of items that should be included in reports of ***cross-sectional studies***

	Item No	Recommendation
**Title and abstract**	1	Cross-sectional Analysis:Waist-to-Hip Ratio and Oxygen Saturation Association in Men Exposed to Long-Term Chronic Intermittent Hypobaric Hypoxia
		The abstract includes:<br>➢ The observational study evaluated the association between waist-to-hip ratio (WHR) and oxygen saturation (SpO_2) in miners chronically exposed to long-termchronic intermittent hypobaric hypoxia.<br>➢ A cross-sectional assessment was conducted in 120 healthy male miners working above 4400 m under a rotational shift system.<br>➢ Anthropometric, physiological, and biochemical measurements were obtained at altitude, including oxygen saturation, waist-to-hip ratio, blood pressure, hematological variables, metabolic biomarkers, and lifestyle factors. Group comparisons were performed using appropriate parametric or non-parametric statistical tests.<br>➢ Participants with WHR > 0.94 exhibited significantly lower oxygen saturation compared with those without cardiometabolic risk (88.8 ± 0.54 vs. 90.41 ± 0.50; p = 0.031).<br>➢ These findings suggest that increased central adiposity may be associated with reduced oxygenation in individuals exposed to long-term intermittent hypobaric hypoxia, highlighting the importance of anthropometric monitoring in occupational high-altitude populations.
**Introduction**		
Background/rationale	2	The introduction describes:<br>➢ The physiological consequences of CIHH exposure including reduced oxygen availability and cardiometabolic alterations.<br>➢ WHR is presented as an indicator of central adiposity potentially influencing oxygenation and metabolic risk in hypoxic environments.
Objectives	3	Determine the association between waist-to-hip ratio (WHR), biomedical variables, and anthropometric variables in men under conditions of long-term CIHH.<br>Hypothesis:<br>Higher waist-to-hip ratio (WHR) values are associated with lower peripheral oxygen saturation in men exposed to long-term chronic intermittent hypobaric hypoxia.
**Methods**		
Study design	4	The study is described as:<br>➢ A cross-sectional observational design was used to evaluate anthropometric, physiological, and biochemical variables measured simultaneously in miners working at high altitude.
Setting	5	Location:<br>➢ The study was conducted in a mining settlement in northern Chile located above 4,400 m. Workers follow a rotating schedule of 7 day high-altitude and 7day sea level. Measurements were performed at the mine health facility approximately 18 hours after arrival at altitude.
Participants	6	Participants:<br>➢ Participants were 120 healthy Chilean male miners randomly selected from workers undergoing occupational health evaluations.<br>➢ Inclusion criteria included more than 5 years of CIHH exposure and absence of major chronic diseases.<br>Exclusion criteria:<br>➢ Diabetes<br>➢ Hypertension<br>➢ Sleep apnea (OSA)<br>➢ Cardiopulmonary disease<br>➢ Supplemental oxygen use<br>Ethics:<br>➢ Approved by Universidad Arturo Prat ethics committee<br>➢ Informed consent obtained

Variables	7	Outcome variable:<br>➢ The primary outcome was oxygen saturation (SpO_2).<br>Exposure variable:<br>➢ The main exposure variable was waist-to-hip ratio (WHR).<br>Additional variables:<br>➢ BMI<br>➢ Blood pressure<br>➢ Heart Rate<br>➢ Hematological parameters<br>➢ Lipid profile<br>➢ Smoking status
Data sources/ measurement	8*	➢ SpO_2 and heart rate were measured using a finger pulse oximeter.<br>➢ Blood pressure was measured with calibrated sphygmomanometers.<br>➢ Anthropometric measurements were performed using standardized procedures.<br>➢ Laboratory variables were obtained using routine clinical assays.<br>* Two measurements averaged for BP and SpO_2.
Bias	9	Potential bias was minimized by applying strict inclusion/exclusion criteria, using standardized measurement procedures, and restricting the study population to healthy workers without cardiopulmonary or metabolic disease.
Study size	10	Sample size:<br>➢ The final analytical sample consisted of 120 miners who met the eligibility criteria and had complete waist-to-hip ratio (WHR), physiological, and anthropometric measurements available for analysis.<br>➢ Standardized effect size (Cohen's d = 0.46) and the corresponding group sizes (n = 45 and n = 48), the estimated statistical power at a two-sided α = 0.05 was 59.2%, indicating limited-to-moderate power to detect differences of this magnitude
Quantitative variables	11	Continuous variables were analyzed using descriptive statistics. WHR was categorized using a threshold of 0.94 to define cardiometabolic risk groups.
Statistical methods	12	(*a*) Statistical analyses included descriptive statistics, Shapiro–Wilk normality tests, Student's t-tests, and Wilcoxon non-parametric tests when appropriate. Analyses were performed using R with significance defined as $p<0.05$.
		(*b*) Comparative analyses were performed between WHR-defined cardiometabolic risk groups.
		(*c*) Missing data were handled using available-case (complete-case) analysis. After enrollment, two participants were excluded, 28 lacked waist-to-hip ratio (WHR) data, and 8 lacked oxygen saturation (SpO_2) measurements. Consequently, each analysis was conducted using the subset of participants with complete data for the variables under evaluation.
		The relationship between WHR and SpO_2, we evaluated distributional patterns, dispersion, and linearity using a scatter plot with fitted regression line and 95% confidence band. Influence diagnostics were conducted using Cook's distance to detect potentially influential observations.
		WHR was rescaled so that one unit corresponds to a 0.1-unit increase (WHR/0.1), allowing direct interpretation of coefficients per 0.1 increase in WHR. We report regression coefficients (β) with 95% confidence intervals and p-values; robust (HC3) standard errors were used when heteroscedasticity was detected.
**Results**		
Participants	13*	A total of 120 miners met eligibility criteria and completed all assessments.
Descriptive data	14*	(a) Participants had a mean age of 41.7 years and mean CIHH exposure of approximately 14 years. Mean BMI was 26.2 kg/m² and 34.8% were smokers
Outcome data	15*	Mean oxygen saturation among participants was 89.07%, reflecting physiological adaptation to chronic hypobaric hypoxia.
Main results	16	(*a*) Participants with WHR >0.94 exhibited significantly lower oxygen saturation compared with those without cardiometabolic risk.

Other analyses	17	No significant differences were observed between WHR groups for blood pressure, heart rate, or years of CIHH exposure.
**Discussion**		
Key results	18	The study demonstrates a significant association between increased central adiposity and reduced oxygen saturation among workers exposed to long-term CIHH.
Limitations	19	➢ The study design does not allow causal inference; therefore, the observed associations should be interpreted as descriptive rather than causal.<br>➢ Selection bias and healthy worker effect: Because workers with cardiopulmonary disease, obstructive sleep apnea, reduced tolerance to high altitude, or other relevant health conditions are routinely excluded from employment and were also excluded from this study, the hematological and physiological profile likely represents a healthier subset of the workforce.<br>➢ Limited external validity: The cohort included only healthy adult male miners, which restricts the generalizability of the findings to women, older individuals, workers with comorbidities, or populations with different genetic and environmental backgrounds.<br>➢ Measurement-related constraints: Although assessments were standardized, acute acclimatization effects and the inherent variability of pulse oximetry at high altitude cannot be completely ruled out.<br>➢ Residual confounding: Lifestyle factors, respiratory mechanics, physical activity, and cardiorespiratory fitness were not directly or comprehensively measured; therefore, mechanistic interpretations remain hypothesis-generating, and unmeasured confounding may have influenced the observed associations.<br>.
Interpretation	20	Using a cross-sectional approach, this study evaluated the relationship between oxygen saturation and waist-to-hip ratio in men exposed to long-term CIHH. Lower SpO_2 was found to be associated with higher WHR; however, this relationship must be interpreted as correlational, as the study design does not allow conclusions regarding directionality or causality. These findings add to the growing body of evidence indicating that oxygenation status and central adiposity are interrelated in high-altitude occupational settings, reinforcing the relevance of monitoring both parameters in this population.
Generalisability	21	Although the adjusted association was modest and imprecise, even small differences in SpO_2 may be operationally meaningful at ~4,400 m, where baseline oxygenation is already reduced. While the clinical implications should be considered cautiously, the observed association provides a basis for future research and supports the potential development of preventive strategies and occupational surveillance programs. Prospective studies with broader physiological characterization will be necessary to determine whether WHR-based thresholds can improve risk stratification in CIHH-exposed workers.
**Other information**		
Funding	22	The APC was funded by Atacama University project: “Fondo Concursable de Apoyo a las Publicaciones Científicas (APC) de la Universidad de Atacama”, and the work of Mário de Castro is partially funded by CNPq, Brazil (grant 301596/2025-5).

*Give information separately for exposed and unexposed groups.

Note: An Explanation and Elaboration article discusses each checklist item and gives methodological background and published examples of transparent reporting. The STROBE checklist is best used in conjunction with this article (freely available on the Web sites of PLoS Medicine at http://www.plosmedicine.org/, Annals of Internal Medicine at http://www.annals.org/, and Epidemiology at http://www.epidem.com/). Information on the STROBE Initiative is available at www.strobe-statement.org.
